# Powder Self-Emulsifying Drug Delivery System for Mitotane: In Vitro and In Vivo Evaluation

**DOI:** 10.3390/pharmaceutics16091194

**Published:** 2024-09-11

**Authors:** Mohamed Skiba, Valentin Lefébure, Frederic Bounoure, Nicolas Milon, Michael Thomas, Herve Lefebvre, Lahiani-Skiba Malika

**Affiliations:** Normandie Univ., UNIROUEN, INSERM, NORDIC UMR 1239, F-76000 Rouen, France; v.lefebure@outlook.fr (V.L.);

**Keywords:** mitotane, Lysodren^®^, α-cyclodextrin, adrenocortical carcinoma, Cushing’s disease, bioavailability, powder self-emulsifying drug delivery system

## Abstract

Drug Delivery Systems (DDSs) of known drugs are prominent candidates for new and more effective treatments of various diseases, as they may increase drug solubility, dissolution velocity, and bioavailability. Mitotane (o,p′-dichlorodimethyl dichloroethane [o,p′-DDD]) is used for the treatment of adrenocortical cancer and, occasionally, Cushing’s syndrome. However, the efficacy of mitotane is limited by its low oral bioavailability, caused by its extremely poor aqueous solubility. This research explores the development of a new powder self-emulsifying drug delivery system (P-SEDDS) for mitotane to improve its oral bioavailability. The study focuses on the new concept of a mitotane-loaded P-SEDDS to overcome the challenges associated with its limited solubility and high logP, thereby improving its therapeutic efficacy, reducing off-target toxicity, and avoiding first-pass metabolism. The P-SEDDS formulations were meticulously designed using only α-cyclodextrin and oil, with the goal of achieving a stable and efficient P-SEDDS. The optimized formulation was characterized for pharmaceutical properties, and its pharmacokinetic behavior was examined in rats. The results demonstrated a significant enhancement in the bioavailability of mitotane when delivered through the P-SEDDS, attributed to the increased dissolution velocity and improved absorption of the poorly water-soluble drug. The results suggest that a mitotane-loaded P-SEDDS has distinctly enhanced in vitro and in vivo performance compared with conventional mitotane formulations (Lysodren^®^), which leads to the conclusion that the P-SEDDS formulation could be a viable and effective strategy for improving the dissolution rate and bioavailability of poorly aqueous-soluble ingredients.

## 1. Introduction

Adrenocortical carcinoma (ACC) is a rare cancerous tumor that develops at the level of the adrenal cortex [1,2]. The annual incidence is estimated at 0.7 to 2 new cases per million inhabitants per year [3], responsible for 0.04 to 0.2% of deaths due to cancer. ACC most often occurs in adults between 40 and 50 years old but also in children under 15 years old. This tumor is more often observed in women than men, without knowing the reason, but the prognosis of this disease is poor because it is diagnosed late, and its medical treatment is very ineffective, with a 5-year survival lower than 40% of cases [4]. Total surgical excision of the tumor represents the best chance of a total cure. Adjuvant treatment may be offered in addition to surgery. This consists of the oral administration of mitotane, o′,p′-DDD (ortho,para′dichloro-diphenyl-dichloroethane), a derivative of the insecticide DDT (dichlorodiphenyltrichloroethane), and the only one approved for ACC. Similarly, in the inoperable advanced forms of the pathology, only mitotane is proposed, because it remains to date the only drug with partial cytotoxic efficacy for the treatment of adrenocortical tumors. A pharmaceutical brand (Lysodren^®^) has been the subject of a European Marketing Authorization since 2004 and a Marketing Authorization in the USA since 1970 after the first clinical study carried out by the R. H. Moy team in 1960 [5], with the official indications being “the treatment of ACC in advanced forms in inoperable patients, in metastatic forms, or in recurrent forms” and palliative treatment in the event of advanced disease.

Mitotane can also be used in Cushing’s disease, a pathology caused by an adrenocorticotropic hormone (ACTH)-secreting pituitary tumor that is the most common cause of excessive endogenous cortisol secretion [6,7,8]. Hypercortisolism can lead to significant morbidity and premature death compared to the general population [9]. The primary goals of Cushing’s disease treatment are to normalize cortisol levels and reverse the signs and symptoms of hypercortisolism [7,8]. The first-line treatment is trans-sphenoidal surgery [7], although this is not always successful [10], and patients can relapse several years after apparent surgical success [11]. A number of medical therapies are currently used in clinical practice for the treatment of Cushing’s disease. These comprise mitotane (an adrenolytic agent), pasireotide (an analog of somatostatin), cabergoline (a dopamine receptor agonist), metyrapone and ketoconazole (inhibitors of adrenal steroidogenesis), and mifepristone (a glucocorticoid receptor antagonist). Since not all patients with Cushing’s disease derive sufficient benefit from available treatments, new formulations are always needed.

**Figure 1:**
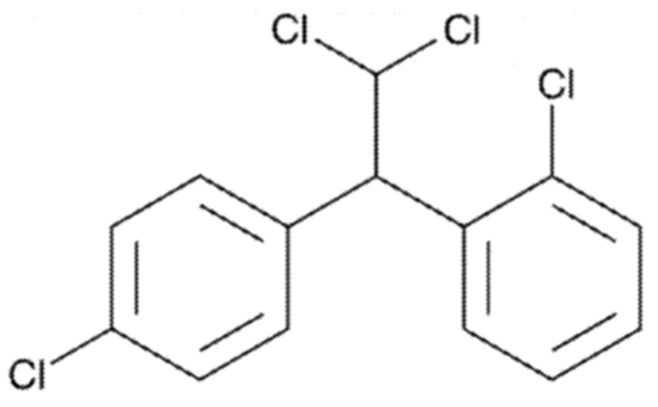
Chemical structure of mitotane. Molecular formula: C_14_H_10_Cl_4_. Molecular weight: 320.04 g/mol.

Mitotane is therefore a molecule of interest, as it is involved in the treatment of two rare diseases. However, mitotane has one drawback: it has very low water solubility (solubility: 0.1 mg/L at 25 °C, logP = 6; Figure 1). This low solubility, coupled with a 35–40% absorption per os, makes mitotane a class IIb molecule in the biopharmaceutical classification system [12,13]. To be effective, the plasma mitotane concentration must reach a therapeutic range between 14 and 20 mg/L [14,15]. Due to solubility and absorption limits, it is estimated that, at a dose of 6 g/d (12 tablets per day), only 45% of patients reach the target concentration within three months [16]. This latency time is due, at least in part, to the fact that mitotane preferentially accumulates in fat at concentrations that could represent 200 times that of plasma, thus decreasing its bioavailability and its therapeutic efficacy. High doses of mitotane have consequences for patients: from 5 mg/L, digestive disorders can be observed—anorexia, nausea, or vomiting and, in some cases, diarrhea. Central nervous system disorders may also occur in 40% of patients: ataxia, confusion, fatigue, dizziness, paresthesia, and polyneuropathy. Recently, oral and vulvo-vaginal lichenoid reactions and encephalopathies have also been reported [16,17,18].

To improve the current therapeutic options for ACC with fewer toxic effects, the development of new formulations containing mitotane or new compounds containing its metabolites should be considered.

In recent years, Drug Delivery Systems (DDSs) have benefited from novel research and the resulting innovation in the field of pharmaceutical sciences. DDSs have obvious advantages, including good pharmacological effectiveness and low toxicity. Diverse formulation methods have been developed to modify the solubility of drugs, such as cyclodextrins, nanocrystals, emulsions, solid dispersion, and lipid-based drug delivery systems. Self-emulsifying drug delivery systems (SEDDSs) are defined as isotropic mixtures of lipid, surfactant, cosurfactant, and drug that rapidly form a microemulsion when mixed with water. Such systems are described in greater detail in Grove and Müllertz [19]. SEDDSs enhance drug delivery by promoting the absorption of drugs via the lymphatic transport pathway, preventing hepatic first-pass metabolism, reducing the effects of intestinal excretion transport, and avoiding the degradation of drugs in the physiological environment. Among them, the P-SEDDS, a novel isotropic system, consists of an oil phase and cyclodextrins, which offer high potential for ameliorating the solubility, dissolution rate, and oral bioavailability of hydrophobic agents [20].

Accordingly, the aim of the current study was to develop a mitotane-loaded Powder SEDDS (mito P-SEDDS) formulation to improve the pharmacokinetic behavior. The selected formulation was evaluated by in vitro dissolution and in vivo studies. In vivo evaluation of the selected formulation was studied in Sprague-Dawley rats, and to assess the relative bioavailability, pharmacokinetic parameters were compared with the conventional mitotane formulation (Lysodren^®^) and the pure drug.

## 2. Material and Methods

### 2.1. Materials

Mitotane (commercialized as racemic mixture), IS (4,4′-DDT), Cremophor^®^ EL, and Tween^®^ 80 were purchased from Sigma-Aldrich (Saint-Quentin-Fallavier Cedex, France), and corn oil was purchased from Cooper (Melun, France). Mitotane tablets (Lysodren tablets) were purchased from HRA rare disease (Paris, France). All solvents used in the HPLC analysis were of HPLC grade purchased from Sigma-Aldrich (Saint-Quentin-Fallavier Cedex, France). All other chemical and analytical reagents were of analytical grade and used as received.

### 2.2. Methods

#### 2.2.1. Preparation of a Mitotane-Loaded P-SEDDS

The mitotane-loaded P-SEDDS is prepared using a patented manufacturing process (PCT/FR2898817A1) [20]. The method of preparing the mitotane-loaded P-SEDDS comprises the solubilization of mitotane in an oil phase with or without a co-solvent, the addition of cyclodextrin in the oil phase with or without an absorption promoter, the addition of the aqueous phase to obtain an O/W primary emulsion then a P-SEDDS, and the drying and grading of the granules containing proportions of oil, cyclodextrin, and mitotane lower than 50%, lower than 60%, and higher than 6%, respectively.

- Preparation of a mitotane-loaded P-SEDDS (Mito F1):

In one step, 25 mL of corn oil mixture loaded with 5 g of mitotane and 3 mL of ethanol as a co-solvent are introduced into a planetary mixer (Hobart type) to improve the solubility of the mitotane in the oil phase. Then, 42.5 g of α-cyclodextrins dispersed in the oily phase are added under stirring (variator No. 1) and at room temperature (25 °C). The mitotane-loaded P-SEDDS is formed after adding an aqueous phase (5 mL of water) under stirring (variator No. 2). The wet granules are then calibrated (1 µm mesh) in an oscillating granulator, then dried in an oven at 45 °C for 15 min until a moisture content of 5% to 6% and elimination of alcohol are obtained. Granules with an average size of 800 µm loaded with 6.8% of mitotane are obtained.

- Preparation of a mitotane-loaded P-SEDDS with absorption promoter (Mito F2):

The operation is carried out as described in the last point, but using 1 mL of Cremophor EL^®^ as the mitotane absorption promoter. Granules with an average size of 800 µm loaded with 6.8% mitotane are obtained.

#### 2.2.2. In Vitro Drug Release

##### Dissolution Studies

Dissolution testing of each batch was performed using a USP II (paddle) apparatus (Copley DIS6000 Dissolution Tester, Copley Scientific Ltd., Nottingham, UK). Dissolution testing was performed using 1000 mL of 0.4% *w*/*v* Span 80 in water at 37 °C, which was added into the solution to approach sink conditions, with a paddle speed of 100 rpm. One tablet or granules of mitotane were tested per dissolution vessel. Samples (each 10 mL) were collected at 5, 10, 15, 20, 25, 30, 35, 40, 45, 50, and 60 min after dropping the tablets, and the same volume of purified water was immediately refilled. The collected samples were immediately filtrated through membrane filters (mixed cellulose ester type membrane filter, pore size: 0.45 μm, 13 mm in diameter). The drug concentrations of the samples were determined by a UV-HPLC method described below.

##### Drug Release Mathematical Modeling

Differences between the release profiles obtained using various dissolution tests were observed. These results suggest the necessity of knowledge about the underlying in vivo processes and the need to translate this research to in vitro test systems [21].

The obtained release profiles were analyzed with zero-, first-, and second-order kinetics, as well as the Korsmeyer–Peppas [22,23], Higuchi [24], and Hixon–Crowell models [25].

The zero-order kinetics model is as follows:

F_t_ = F_0_ + Ϗ_0_ × t (1)

where F_t_ is the amount of drug released or dissolved over time t, F_0_ is the initial amount of drug in solution (it is usually zero), and Ϗ_0_ (Kappa) is the zero-order release rate constant

The first-order kinetics model is as follows:

Log F_t_ = Log F_0_ − Ϗ_1_t/2.303 (2)

where F_0_ is the initial concentration of drug, F_t_ is the concentration of drug in solution at time t, and Ϗ_1_ is the first-order release rate constant.

The Korsmeyer–Peppas model [22,23,26] is as follows:

F_t_/F_α_ = Ϗ_KP_ × t_n_ (3)

where F_t_/F_α_ is the fraction of drug released at time t, Ϗ_KP_ is the rate constant (having units of t_n_), and n is the parameter indicative of the drug release mechanism.

The Higuchi model [24] is as follows:

F= Ϗ_H_ × t^½^ (4)

where F is the amount of drug released in time t and Ϗ_H_ is the Higuchi rate constant.

The Hixon–Crowell model [25] is as follows:

F_0_^1/3^ − F_t_^1/3^ = Ϗ_HC_ × t (5)

where F_0_ is the initial amount of drug in the pharmaceutical dosage form, F_t_ is the remaining amount of drug in the pharmaceutical dosage form at time t, and Ϗ_HC_ (Kappa) is the Hixon–Crowell rate constant.

Linear regression analysis based on the least-squares regression method was employed to study the linearity of the kinetic models. Comparing the standard deviation (SD) and the correlation coefficient R^2^ allowed us to choose the kinetic model that describes the observed processes well. Statistical analysis using an analysis of variance (ANOVA) with Tukey’s test and Student’s *t*-test was used to assess the differences between the obtained release profiles. A statistically significant difference was indicated when *p* < 0.05 [26].

##### UV-HPLC Assays

The determination of mitotane was carried out using HPLC (Agilent HPLC 1100; Agilent Technologies, Waldbronn, Germany). Twenty microliters of sample were injected onto a C_18_ column (Nucleosil^®^, 5 μm, 0.46 mm, 25 cm; Macherey Nagel, Eckbolsheim, France) using an autosampler (G1329A). The mobile phase was a mixture of methanol and water (85:15, *v*/*v*) at a flow rate of 1.5 mL/min (G1312A). The detection was performed with UV spectrophotometry at 230 nm (VWD G1314B), and peak surface was used for the quantification.

#### 2.2.3. Pharmacokinetics Profile

##### In-Vivo Study and Pharmacokinetic Analysis

- GLP regulation requirements

The study took place according to the guidelines concerning Good Laboratory Practice (GLP) dated 14 March 2000 and published by the French Ministry of Social Affairs and National Solidarity and the State Secretariat for Health, which are in accordance with Directive 2004/10EC and are accepted by the US FDA and Japanese authorities.

The study also took place according to the consensus document Management of Multi-site Studies: ALS190407 (Test facility: Avogadro LS Parc de Génibrat 31470 Fontenilles, France).

The study plan was favorably assessed by the Avogadro LS Animal Ethics Committee.

- Animals

Twenty-four Sprague-Dawley male rats (Charles River, BP 010969592 L’Arbresle, France) weighing 200–250 g were used. The animals were housed in individual cages with ad libitum food and water. Environmental enrichment was supplied for each animal. None of them was medicated during the trial. A daily light cycle of 12 h day/12 h night was applied in the housing room.

- Protocol

The animals were divided into three groups: the first one received Lysodren^®^ (Animals ID1 to 8), the second one received a MitoF1 formulation (Animals ID9 to 16), and the last one a MitoF2 formulation (Animals ID17 to 24). In each group, the formulation was administrated by oral gavage in the form of an aqueous suspension (Lysodren^®^ tablets were ground to obtain a powder) at a dose of 100 mg/kg or 10 mL/kg at T0. Blood specimens, 0.2 mL, were drawn via femoral catheter into Lithium Heparin tubes on the eve of dosing (Predose), and then at 30 min, 1 h, 2 h, 3 h, 4 h, 5 h, 6 h, 9 h, 12 h, 24 h, and 48 h post dose. The animals were weighed on each dosing day. All animals were euthanized after the last blood sampling (except rat number 17, which died 30 minutes after the first blood sampling). The aim of this test was to study the pharmacokinetic characteristics of three different formulations containing mitotane. Non-compartmental analysis of individual plasma concentrations was undertaken using Phoenix^®^ WinNonlin^®^ 8.1 (Certara, Radnor, PA, USA).

##### HPLC Analysis

- Chromatographic conditions

The dosage was determined using an internal standard (IS): 4,4′-DDT. The equipment used for HPLC is referenced in Table 1. A BEH C18 100 × 2.1 mm, 1.7 µm column, maintained at 40 °C (±5 °C) with a 0.2 µm guard column was used. A mixture of water (mobile phase A) and acetonitrile (mobile phase B) was used for the mobile phase (Table 1). The retention time for mitotane using this technique was 4.20 min, and for the IS it was 4.75 min. A 10 µL volume of sample was injected into the mobile phase, and then detected by UV at 230 nm. The ULOQ and LLOQ were determined at respective concentrations of 10,000 ng/mL and 50 ng/mL.

- Validation method

The objective of this delegated phase was to develop and qualify an LC-UV method for the assay of mitotane in rat plasma samples with a target LLOQ of 50 ng/mL, and to assay mitotane in the rat plasma samples from the PK study.

##### Statistical Analysis

Statistical analyses and the creation of figures were conducted using R^2^ (version 4.3.3; 29 February 2024). As the data were unpaired and non-normal, the non-parametric Wilcoxon Mann–Whitney test was used.

The results shown represent the average values with a standard deviation (SD) derived from four determinations for the C_max_, AUC_0–t_, AUC_inf_, t_1/2_, and Lz comparisons.

## 3. Results and Discussion

### 3.1. Dissolution

The results section presents the findings of the study, including the prepared Mitotane-loaded P-SEDDS formulation and its characterization data. The in vitro drug release studies revealed a significant enhancement in the dissolution profile of mitotane–loaded P-SEDDS compared to Lysodren^®^ and to the pure drug.

**Figure 2:**
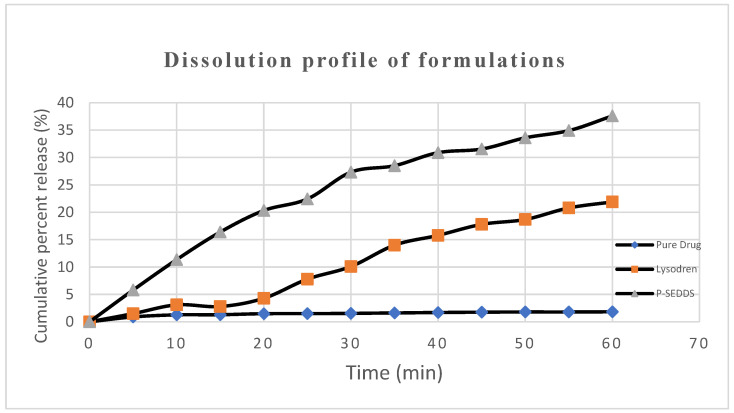
Cumulative percent of release of mitotane from pure drug, Lysodren^®^ and P-SEDDS; data expressed as mean (*n* = 3).

The dissolution profiles of the mitotane–loaded P-SEDDS, Lysodren^©^, and the pure drug are shown in Figure 2. To compare the dissolution performance, the profiles are plotted using the percentage of the mass of mitotane dissolved, i.e., the mass dissolved after the infinity spin., 

The mitotane-loaded P-SEDDS formulation exhibited faster drug release characteristics (>20%) within 20 min. On the contrary, the Lysodren^®^ formulation showed a maximum 10% release only in a 30 min time period. A nearly 5-fold (in 20 min) improvement in the dissolution rate was, therefore, revealed by the mitotane-loaded P-SEDDS formulations vis-à-vis the Lysodren^®^ formulation. The observations revealed from the present studies are in consonance with the previously published literature reports about a self-emulsifying drug delivery system strategy and about the pure drug [27,28].

The release profiles were fitted to five different kinetic models to analyze the in vitro release kinetics. The drug-loaded P-SEDDS was considered a water-activated release system, considering the drug’s uniform distribution and amorphousness in P-SEDDS. R^2^ is a goodness of fit parameter; higher values indicate better model fitting.

**Figure 3:**
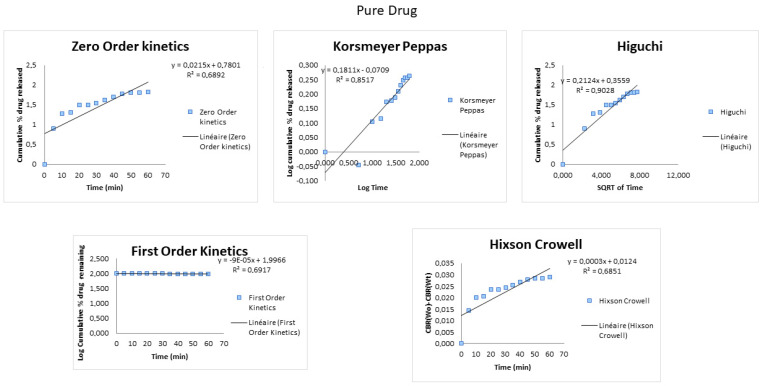

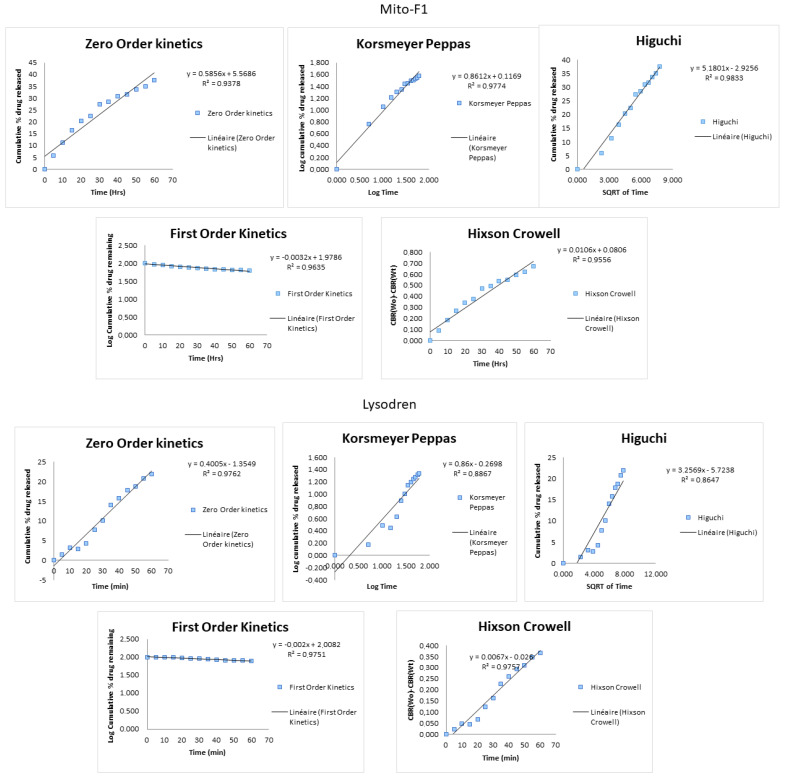
Drug release data fitted to various kinetic models (zero-order, first-order, Korsmeyer–Peppas, Hixson–Crowell, and Higuchi) obtained from the dissolution studies of pure drug, Lysodren^®^, and MitoF1.

The dissolution data up to 60 min of drug release for MitoF1, Lysodren^®^, and the pure drug were fitted to the zero order, first order, Korsmeyer–Peppas, Hixson–Crowell, and the Higuchi drug diffusion models, as can be seen in Figure 3. In Table 2, the obtained correlation coefficients (R^2^) for the different models are displayed. The pure drug and MitoF1 showed a high R^2^ for the Higuchi drug diffusion model, unequivocally indicating the drug release from MitoF1 to be governed via the Fickian diffusion mechanism. This can be explained owing to the immediate drug release characteristics of the Lysodren^®^ formulation, without depicting any lag time, which indicates that the drug is released from the dosage form by diffusion, and that the release is time-dependent.

The drug release of the Lysodren^®^ tablets showed good correlation (R^2^ = 0.9762) for the zero order. This relationship can be used for dosage forms that do not disaggregate and release the drug slowly, independent of drug concentration.

### 3.2. Pharmacokinetics Profile

#### 3.2.1. Method Validation

Full details of the HPLC method and validation are given in Section 1 of the Supporting Information.

The method was validated according to the ICH guidelines on validation of analytical procedures [29]. The detection wavelength, 230 nm, was determined by scanning the maximum absorbance wavelength of mitotane (2,4′-DDD) and the internal standard (4,4′-DDT) in the mobile phase using a UV spectrophotometer. The specificity of the method was determined by analyzing 13 different blank rat plasma samples to demonstrate lack of chromatographic interference from endogenous plasma components at the retention time of both mitotane and the internal standard (IS) (Supplementary S1 Figure S1). No significant interfering peak from plasma endogenous compounds was observed for the retention times of mitotane and the internal standard, which were 4.22 min and 4.74 min, respectively (Supplementary S1 Figure S2). The limit of detection (LOD) and lower limit of quantitation (LLOQ) were determined from the ratio of peak signal and baseline noise and were 40 ng/mL and 50 ng/mL, respectively, for this method (Supplementary S1 Figure S3). The linearity of the calibration curve for mitotane was assessed over the range of 50 to 10,000 ng/mL (Supplementary S1 Figure S4) and was performed after subjecting plasma samples to the sample preparation procedure followed by injection onto the HPLC system. A standard curve was constructed by plotting the peak area ratios of mitotane and the internal standard against the mitotane concentrations in the plasma. The results were fitted to linear regression analysis. Six replicates of the calibration curve were prepared, taking each concentration six times. The regression coefficient (R^2^) value was used to evaluate the linearity of the calibration curve. The standard curves of mitotane in rat plasma were linear with a reliable reproducibility over the ranges of 50 to 10,000 ng/mL, and the regression coefficients (R^2^) were over 0.993109 from each standard curve of six separate runs. The accuracy and precision were confirmed by analyzing six replicates at three QC levels (low, medium, and high) and the LLOQ.

The data confirmed the satisfactory accuracy, precision, and reproducibility of the method. In the pharmacokinetic analysis, the residual method was adapted to estimate the pharmacokinetic parameters of the three treatments for each subject (Supplementary S1 Table S4).

#### 3.2.2. In Vivo Study and Pharmacokinetic Analysis

**Figure 4:**
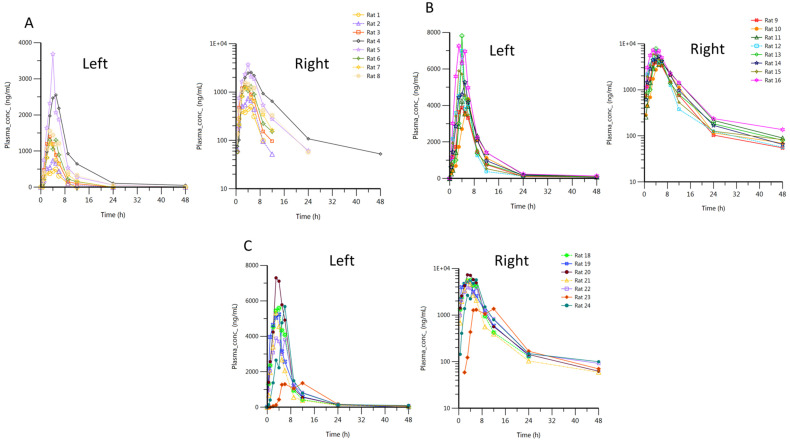
Individual plasma concentration of mitotane after oral administration of (**A**) Lysodren^®^, (**B**) MitoF1 and (**C**) MitoF2 at 100 mg/kg in rat in linear scale (**left panel**) and semi-log scale (**right panel**).

The pharmacokinetic parameters of mitotane following oral administration of single doses of 100 mg kg-1 of (A) Lysodren^®^ tablets and (B) Mito-PSEDDS with (MitoF1) and without Cremophor EL^®^ (MitoF2) into rats are shown in Table 3. Moreover, the mitotane plasma concentration time profiles of both treatments are depicted in Figure 4 and could best be described by a non-compartmental model.

Figure 4 illustrates the individual plasma concentration versus time profile of mitotane from the Lysodren^®^, MitoF1, and MitoF2 formulations (Supplementary S2 Table S11). As per the chosen non-compartmental body model, various pharmacokinetic parameters were computed, which revealed markedly superior drug absorption rates, with distinct improvement in C_max_, T_max_ and AUC_last_ for MitoF1 and MitoF2 vis-à-vis Lysodren^®^.

We can observe a strong improvement of mitotane’s pharmacokinetics with the MitoF1 and MitoF2 formulations in comparison with the Lysodren^®^ form. Indeed, C_max_ is 3 times greater for MitoF2 and 3.5 times greater for MitoF1 than for Lysodren^®^. If we look at the AUC reflecting the plasma exposure, we can see that it is 3.5 times higher for MitoF2 and 4.2 times higher for MitoF1 than for Lysodren^®^. We can correlate this last information with a decreased elimination rate constant by a factor of 2.3 for MitoF2 and by 2.5 for MitoF1. In the case of t_1/2_, it is 2.4 times higher for MitoF2 and 2.8 for MitoF1. In contrast, we do not observe any difference in T_max_ for each formulation.

We could notice tolerance problems for two rats of MitoF2: respiratory difficulties after administration were observed.

Overall, the studies revealed supremacy in the rate and extent of drug absorption from the Mito-PSEDDS over Lysodren^®^. Interestingly, no significant difference in pharmacokinetic parameters was observed between MitoF1 and MitoF2. This means that the formulation of P-SEDDS helped improve the formulation characteristics by retaining the in vivo pharmacokinetic performance.

This new P-SEDDS is a major improvement in mitotane’s pharmacokinetics. Indeed, MitoF1 and MitoF2 increase the bioavailability of our active principal. In comparison with the work of Attivi et al. [28] on a new oral form of mitotane using a liquid self-emulsion system, we also found an increase in our C_max_, an unchanged T_max_, and an increase in AUC_inf_. However, the C_max_ found is much higher (5.711 mg/L for MitoF1 and 4.911 mg/L for MitoF2 versus 2.2 mg/L). The increases in the AUC_inf_ were also greater, particularly for the MitoF1 form, with an increase by a factor of 3.84 versus 3.4. However, some of these differences can be explained by the difference in model used (rat vs. rabbit) and the structure of the self-emulsifying system used, which was liquid and not in powder form in Attivi’s study, because of the wide inter-individual variability in mitotane metabolism [16,30,31,32,33].

We can ask about the tolerance of Cremophore EL^®^ or the interaction mitotane/Cremophor EL^®^ because no side effects were reported for the rats medicated by Lysodren^®^ and MitoF1, but 25% of the rats treated with MitoF2 had respiratory difficulties (for rat number 17, the plasma concentration of this animal at 30 min was about 15-fold higher than that of the other animals in the same group; this high concentration could explain its death). These side effects can also be explained by the difference in metabolism between specimens; in fact, the difference in body fat and the metabolism profile (slow, normal, or fast) can change the mitotanemia.

Clinical studies are needed to complete this study in order to analyze the safety of the Mito-PSEDDS in a human model. Moreover, if the augmentation of absorption is validated for humans, we can assume that the management of patients requiring mitotane-based treatment will be greatly improved. Indeed, with a short time to reach the 14–20 mg/L range, the treatment’s onset of action will be shortened. For ACC, we can hope to move from palliative to curative treatment, or at least significantly increase the 5-year survival rate.

### 3.3. Statistical Analysis

Mitotane pharmacokinetic data were compared with a one-way analysis of variance using R^2^ (version 4.3.3; 29 February 2024). As the data were unpaired and non-normal, the non-parametric Wilcoxon Mann–Whitney test was used. A *p*-value < 0.05 was considered to be significant.

**Figure 5:**
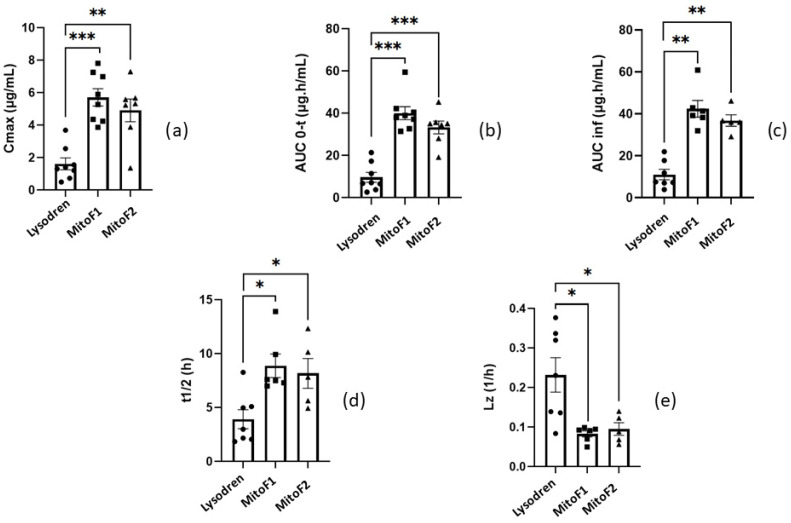
Graphical representation of the significance of: (**a**) C_max_; (**b**) AUC_0–t_; (**c**) AUC_inf_; (**d**) t_1/2_; (**e**) Lz. ***, **, * indicates *p*-value < 0.001, 0.01, and 0.5, respectively.

MitoF1 and MitoF2 showed a significant difference; *p*-value < 0.001 and 0.01, respectively, in C_max_; *p*-value < 0.001 about AUC_0–t_; *p*-value < 0.01 for AUC_inf_; *p*-value < 0.5 in t_1/2_ and Lz (see Figure 5). We could see an increase in C_max_, AUC_0–t_, AUC_inf_, and t_1/2_ and a diminution in Lz in comparison with Lysodren^®^. On the other hand, there was no difference between MitoF1 and MitoF2 on the same data (*p*-value > 0.5) (Table 4, Figure 5).

## 4. Conclusions

We attempted to develop a mitotane-loaded PSEDDS because of the adrenolytic drug mitotane’s unfavorable pharmacokinetic profile of a high logP, poor aqueous solubility, and extremely high daily dosage (up to 6 g per day in divided doses), which may enable improved oral administration formulation to overcome critical clinical limitations. α-cyclodextrin and corn oil were capable of increasing the solubility and bioavailability of mitotane. To the best of our knowledge, no such powder-emulsifying drug delivery system has ever been published in the literature. A remarkable increase in the dissolution rates and bioavailability of mitotane compared to that of the commercial product was obtained. The corn oil and α-cyclodextrin excipients are highly compatible pharmaceutical ingredients. It stands to reason that this PSEDDS, for the first time, might allow the oral administration of mitotane, which will help to alleviate the side effects associated with the use of high-dose oral therapy of 6 g per day, resulting in a more predictable outcome.

The outstanding findings of the current studies, therefore, ratified the successful selection of a PSEDDS as an effective and cost-effective drug delivery strategy for oral bioavailability enhancement of mitotane and other similar BCS class II and class IV drugs for this purpose.

## Figures and Tables

**Table 1:** HPLC equipment and mobile phase during time.

Autosampler	Acquity UPLC Sample Manager FTN with Sample Organizer (Waters, Saint-Quentin-en-Yvelines cedex, France)		
LC pump	Acquity UPLC I-Class (Waters)		
Column oven	Acquity UPLC Column Heater (Waters)		
Detector	Acquity PDA Detector (Waters)		
Software	MassLynx 4.2 & TargetLynx 4.2 (Waters)		
**Time (min)**	**Flow Rate (mL/min)**	**Mobile Phase A (%)**	**Mobile Phase B (%)**
0.0	0.5	60	40
5.0	0.5	10	90
7.0	0.5	10	90
7.1	0.5	60	40
9.0	0.5	60	40

**Table 2:** In vitro release kinetic values.

Formulation	Zero Order ^1^ R^2^	First Order ^2^ R^2^	Higuchi ^3^ R^2^	Hixson-Crowell ^4^ R^2^	Korsmeyer–Peppas ^5^ R^2^
MitoF1	0.9378	0.9635	**0.9833**	0.9556	0.9774
Lysodren^®^	**0.9762**	0.9751	0.8647	0.9757	0.8867
Pure drug	0.6892	0.6917	**0.9028**	0.6851	0.8517

^1^ Zero-order equation: F_t_ = F_0_ + Ϗ_0_ t; ^2^ First-order equation: log F_t_ = log F_0_ – Ϗ_1_t/2.303; ^3^ Higuchi equation: F= Ϗ_H_ × t^½^; ^4^ Hixson–Crowell equation: F_0_^1/3^ − F_t_^1/3^ = Ϗ_HC_ × t; ^5^ Korsmeyer–Peppas equation: F_t_/F_α_ = Ϗ_KP_ × t_n_. R^2^: correlation coefficient; F_t_: amount of drug released in time t; F_0_: initial amount of drug in dosage form; Ϗ_0_, Ϗ_1_, Ϗ_H_, Ϗ_HC_, Ϗ_KP_: release rate constants. The bold data indicate that the drug release of the Lysodren^®^ showed good correlation (R^2^ = 0.9762) for the zero order; the drug release of the Mito-F1 showed good correlation (R^2^ = 0.9833) for the Higuchi; the drug release of the Pure drug showed good correlation (R^2^ = 0.9028) for the Higuchi.

**Table 3:** Individual and mean PK parameters of mitotane in rats (Lysodren® (animals ID 1 to 8), MitoF1 (animals ID 9 to 16) and MitoF2 (animals ID 17 to 24)); not determined or not applicable; **^(1)^** Harmonic mean for t_1/2_ and geometric mean for Lz; **^(2)^** Not reported since R²_adj_ < 0.8.

**Animal ID**	**T_max_** **(h)**	**C_max_** **(µg/mL)**	**AUC_0–t_** **(µg·h/mL)**	**Rsq²**	**t_1/2_** **(h)**	**Lz** **(1/h)**	**Lz Lower** **(h)**	**Lz Upper** **(h)**
1	5	0.4975	2.635	-	-	-	-	-
2	4	0.7330	3.761	0.95	1.839	0.377	5	12.02
3	3	1.399	6.779	0.95	2.055	0.337	4	12
4	5	2.551	21.34	0.82	8.269	0.0838	6	48
5	4	3.684	17.32	0.98	4.977	0.139	8.983	23.98
6	3	1.302	7.103	0.91	2.169	0.320	5	12
7	3	1.212	6.584	1.00	2.997	0.231	6	12
8	3	1.552	11.43	0.99	5.092	0.136	9	24
N	8	8	8	-	7	7	-	-
Mean ^(1)^	-	1.616	9.619	-	2.987	0.204	-	-
SD	-	1.035	6.618	-	-	-	-	-
CV%	-	64	69					
Min	3	0.4975	2.635	-	-	-	-	-
Median	3.5	1.351	6.941	-	-	-	-	
**Animal ID**	**T_max_** **(h)**	**C_max_** **(µg/mL)**	**AUC_0–t_ (µg·h/mL)**	**Rsq²**	**t_1/2_** **(h)**	**Lz** **(1/h)**	**Lz Lower** **(h)**	**Lz Upper** **(h)**
9	4	3.871	31.41	0.81	7.007	0.0989	5	48
10	6	4.350	32.71	0.70	- ^(2)^	- ^(2)^	-	-
11	4	4.243	40.40	0.88	7.608	0.0911	5	48
12	4	6.991	37.19	0.84	13.90	0.0499	12	48.02
13	4	7.815	38.06	0.84	9.935	0.0698	9	48
14	5	5.267	42.2	0.84	7.311	0.0948	6	48
15	3	5.895	38.99	0.75	- ^(2)^	- ^(2)^	-	-
16	3	7.254	59.41	0.82	7.500	0.0924	4	47.98
N	8	8	8	-	6	6	-	-
Mean	-	5.711	40.05	-	8.370	0.0806	-	-
SD	-	1.515	8.631	-	-	-	-	-
CV%	-	27	22	-	-	-	-	-
Min	3	3.871	31.41	-	-	-	-	-
Median	4	5.581	38.53	-	-	-	-	-
**Animal ID**	**T_max_** **(h)**	**C_max_** **(µg/mL)**	**AUC_0–t_ (µg·h/mL)**	**Rsq²**	**t_1/2_** **(h)**	**Lz** **(1/h)**	**Lz Lower** **(h)**	**Lz Upper** **(h)**
18	4	5.590	35.70	0.90	5.646	0.123	9	24
19	4	5.235	35.08	0.96	4.938	0.140	9	24
20	3	7.297	45.27	0.84	10.14	0.0684	9	47.9
21	3	5.325	28.08	0.80	12.33	0.0562	9	47.88
22	3	3.890	35.06	0.81	7.830	0.0885	4	47.87
23	12	1.361	19.19	-	-	-	-	-
24	6	5.678	34.51	0.69	- ^(2)^	- ^(2)^	-	-
N	7	7	7	-	5	5	-	-
Mean	-	4.911	33.27	-	7.277	0.0899	-	-
SD	-	1.856	7.989	-	-	-	-	-
CV%	-	38	24	-	-	-	-	-
Min	3	1.361	19.19	-	-	-	-	-
Median	4	5.325	35.06	**-**	**-**	**-**	**-**	**-**
Max	12	7.297	45.27	-	-	-	-	-

**Table 4:** Wilcoxon Mann–Whitney test *p*-values.

**C_max_**	**Lysodren**	**MitoF1**	**MitoF2**	**AUC_0–t_**	**Lysodren**	**MitoF1**	**MitoF2**
Lysodren		0.0001554	0.00373	Lysodren		0.0001554	0.0006216
MitoF1			0.6126	MitoF1			0.152
MitoF2				MitoF2			
**t_1/2_**	**Lysodren**	**MitoF1**	**MitoF2**	**Lz**	**Lysodren**	**MitoF1**	**MitoF2**
Lysodren		0.01399	0.04798	Lysodren		0.01399	0.04798
MitoF1			0.9307	MitoF1			0.9307
MitoF2				MitoF2			
**AUC_inf_**	**Lysodren**	**MitoF1**	**MitoF2**				
Lysodren		0.001166	0.002525				
MitoF1			0.2468				
MitoF2							

## Data Availability

The original contributions presented in the study are included in the article/Supplementary Material, further inquiries can be directed to the corresponding author.

## Supplementary Materials

The following supporting information can be downloaded at: https://www.mdpi.com/article/10.3390/pharmaceutics16091194/s1, File S1. Validation method; File S2. Individual mitotane plasma concentrations in rats.

## References

[1] Else T., Kim A.C., Sabolch A., Raymond V.M., Kandathil A., Caoili E.M., Jolly S., Miller B.S., Giordano T.J., Hammer G.D. (2014). Adrenocortical Carcinoma. Endocr. Rev..

[2] Fassnacht M., Libé R., Kroiss M., Allolio B. (2011). Adrenocortical carcinoma: A clinician’s update. Nat. Rev. Endocrinol..

[3] Fassnacht M., Kroiss M., Allolio B. (2013). Update in Adrenocortical Carcinoma. J. Clin. Endocrinol. Metab..

[4] Assie G., Antoni G., Tissier F., Caillou B., Abiven G., Gicquel C., Leboulleux S., Travagli J.-P., Dromain C., Bertagna X. (2007). Prognostic Parameters of Metastatic Adrenocortical Carcinoma. J. Clin. Endocrinol. Metab..

[5] Bergenstal D., Hertz R., Lipsett M.B., Moy R.H. (1960). Chemotherapy of adrenocortical cancer with o,p′DDD. Ann. Int. Med..

[6] Lacroix A., Feelders R.A., Stratakis C.A., Nieman L.K. (2015). Cushing’s syndrome. Lancet.

[7] Biller B.M.K., Grossman A.B., Stewart P.M., Melmed S., Bertagna X., Bertherat J., Buchfelder M., Colao A., Hermus A.R., Hofland L.J. (2008). Treatment of adrenocorticotropin-dependent Cushing’s syndrome: A consensus statement. J. Clin. Endocrinol. Metab..

[8] Pivonello R., De Leo M., Cozzolino A., Colao A. (2015). The Treatment of Cushing’s Disease. Endocr. Rev..

[9] Pivonello R., De Martino M.C., De Leo M., Lombardi G., Colao A. (2008). Cushing’s Syndrome. Endocrinol. Metab. Clin. N. Am..

[10] Tritos N.A., Biller B.M.K., Swearingen B. (2011). Management of Cushing disease. Nat. Rev. Endocrinol..

[11] Dimopoulou C., Schopohl J., Rachinger W., Buchfelder M., Honegger J., Reincke M., Stalla G.K. (2013). Long-term remission and recurrence rates after first and second transsphenoidal surgery for Cushing’s disease: Care reality in the Munich Metropolitan Region. Eur. J. Endocrinol..

[12] Hahner S., Fassnacht M. (2005). Mitotane for adrenocortical carcinoma treatment. Curr. Opin. Investig. Drugs.

[13] Igaz P., Tombol Z., Szabo P.M., Liko I., Racz K. (2008). Steroid biosynthesis inhibitors in the therapy of hypercortisolism: Theory and practice. Med. Chem..

[14] Terzolo M., Pia A., Berruti A., Osella G., Alì A., Carbone V., Testa E., Dogliotti L., Angeli A. (2000). Low-dose monitored mitotane treatment achieves the therapeutic range with manageable side effects in patients with adrenocortical cancer. J. Clin. Endocrinol. Metab..

[15] Terzolo M., Zaggia B., Allasino B., De Francia S. (2014). Practical treatment using mitotane for adrenocortical carcinoma. Curr. Opin. Endocrinol. Diabetes Obes..

[16] Haider M.S., Ahmad T., Groll J., Scherf-Clavel O., Kroiss M., Luxenhofer R. (2021). The Challenging Pharmacokinetics of Mitotane: An Old Drug in Need of New Packaging. Eur. J. Drug Metab. Pharmacokinet..

[17] Schmouchkovitch A., Herry H., Thuillier P., Kerlan V., Fleuret C., Le Toux G., Boisramé S. (2017). Oral and vulvo-vaginal lichenoid reactions due to mitotane (Lysodren^®^): A case report. Medicine.

[18] Lung B.Y., Valentino E., Gerst S.R., Untch B.R., Katz S., Strong V.E., Raj N.P., Olino K., Saltz L., Reidy D.L. (2015). Low objective response and high toxicity to single-agent mitotane in patients with metastatic adrenocortical carcinoma (ACC): A 25 year experience at MSKCC. J. Clin. Oncol..

[19] Grove M., Müllertz A., Hauss D.J. (2007). Chapter 5: Liquid Self-Microemulsifying Drug Delivery Systems. Oral Lipid-Based Formulations—Enhancing the Bioavailability of Poorly Water-Soluble Drugs.

[20] Skiba M., Lahiani-Skiba M., Bounoure F., Thomas M., Lefebvre H. (2020). Pharmaceutical Composition Comprising Mitotane Administered Orally for Treatment of Adrenocortical Carcinoma and Cushing’s Syndrome.

[21] Stein S., Auel T., Kempin W., Bogdahn M., Weitschies W., Seidlitz A. (2018). Influence of the test method on in vitro drug release from intravitreal model implants containing dexamethasone or fluorescein sodium in poly(d,l-lactide-co-glycolide) or polycaprolactone. Eur. J. Pharm. Biopharm..

[22] Korsmeyer R.W., Gurny R., Doelker E., Buri P., Peppas N.A. (1983). Mechanisms of solute release from porous hydrophilic polymers. Int. J. Pharm..

[23] Peppas N.A. (1985). Analysis of Fickian and non-Fickian drug release from polymers. Pharm. Acta Helv..

[24] Siepmann J., Peppas N.A. (2011). Higuchi equation: Derivation, applications, use and misuse. Int. J. Pharm..

[25] Hixon A.W., Crowell J.H. (1931). Dependence of Reaction Velocity upon Surface and Agitation. Ind. Eng. Chem..

[26] Costa P., Lobo J.M.S. (2001). Modeling and comparison of dissolution profiles. Eur. J. Pharm. Sci..

[27] Trotta M., Gallarate M., Pattarino F., Morel S. (2001). Emulsions containing partially water-miscible solvents for the preparation of drug nanosuspensions. J. Control. Release.

[28] Attivi D., Ajana I., Astier A., Demoré B., Gibaud S. (2010). Development of microemulsion of mitotane for improvement of oral bioavailability. Drug Dev. Ind. Pharm..

[29] International Conference on Harmonisation of Technical Requirements for Registration of Pharmaceuticals for Human Use ICH Harmonised Triplicate Guideline on Validation of Analytical Procedures: Text and Methodology Q2 (R1). https://database.ich.org/sites/default/files/Q2%28R1%29%20Guideline.pdf.

[30] Siepmann J., Peppas N.A. (2001). Modeling of drug release from delivery systems based on hydroxypropyl methylcellulose (HPMC). Adv. Drug Deliv. Rev..

[31] Corso C.R., Acco A., Bach C., Bonatto S.J.R., de Figueiredo B.C., de Souza L.M. (2020). Pharmacological profile and effects of mitotane in adrenocortical carcinoma. Br. J. Clin. Pharmacol..

[32] Lehmann T.P., Wrzesinski T., Jagodzinski P.P. (2013). The effect of mitotane on viability, steroidogenesis and gene expression in NCI-H295R adrenocortical cells. Mol. Med. Rep..

[33] Lund B.O., Bergman A., Brandt I. (1989). In vitro macromolecular binding of 2-(2-chlorophenyl)-2-(4-chlorophenyl)-1,1-dichloroethane (o,p’-DDD) in the mouse lung and liver. Chem. Biol. Interact..

