# Supplementary material for: Powder Self-Emulsifying Drug Delivery System for Mitotane: In Vitro and In Vivo Evaluation
Source: Pharmaceutics. 2024 Sep 11;16(9):1194. doi: 10.3390/pharmaceutics16091194 (PMC11434810; doi:10.3390/pharmaceutics16091194)
Supplement: Supplementary file 1 [file pharmaceutics-16-01194-s001.zip › pharmaceutics-3136214-supplementary.pdf]

Supplementary Information (S1 & S2)

Powder Self-Emulsifying Drug Delivery System for Mitotane: In Vitro and In Vivo Evaluation

Mohamed Skiba *, Valentin Lefébure, Frederic Bounoure, Nicolas Milon, Michael Thomas, Herve Lefebvre and Lahiani-Skiba Malika

Normandie Univ., UNIROUEN, INSERM, NORDIC UMR 1239, F-76000 Rouen, France

File S1 : Validation method

1. Introduction

The objective of this delegated phase was to develop and qualify an LC-UV method for the assay of mitotane in rat plasma samples with a target LLOQ of 50 ng/mL, and to assay mitotane in rat plasma samples from PK study.

2. Materials and methods

2.1. Analytical standards

The main characteristics of mitotane and 4,4′-DDT, used as internal standard, are presented below.

	Mitotane (2,4′-DDD)	4,4′-DDT
Molecular formula	$C_{14}H_{10}Cl_4$	$C_{14}H_9Cl_5$
Molecular structure	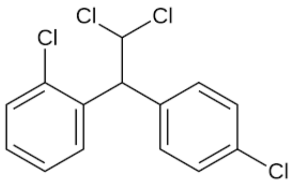	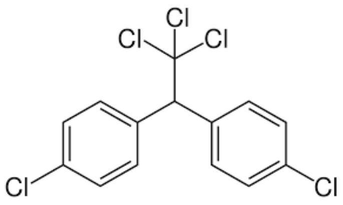
Molecular weight	320.04 g/mol	354.49 g/mol
Supplier	HRA Pharma	Sigma-Aldrich
Batch number	9F05576 (0002350433)	BCBW0671

2.2. Source of control matrix

Control (drug free) Spague Dawley rat plasma samples taken from blood sampled on LH tube were obtained from EAA FTN's stock. Other details concerning the control plasma (named control matrix hereafter) are included in the raw data.

2.3. Development process summary

First, spectrometric conditions (UV and MS/MS using APCI ionization) were optimized in order to obtain the most intensive signal possible for the target LLOQ (50 ng/mL).

According to current knowledge and to bibliographic research, an initial LC-UV method was established:

- Protein precipitation by acetonitrile in rat plasma and reconstitution in a mixture of water and acetonitrile 40/60 (v/v) after evaporation,
- Gradient elution using water and acetonitrile on an Acquity BEH C18 1.7 µm column,
- Comparison of UV and MS/MS (APCI ionization) detection

A summary of the tests performed during development phase and corresponding results, are summarized below in Table S1:

Table S1:

**Analytical run**	**Description**	**Results**
M197400OptA	Optimization of the spectrometric parameters (MS/MS and UV)	Determination of MRM transition in APCI positive ion mode and of UV wavelength
M197400A	Optimization of the chromatographic conditions (mobile phase, gradient programming, injection volume, detection mode)	Gradient elution mode using water and acetonitrile<br>Use of UV detection (APCI MS/MS not sensitive enough)
M197400B	Optimization of protein precipitation (test of acetonitrile and methanol as solvent for precipitation)	Acetonitrile kept as solvent for precipitation (about 90% of extraction recovery, no interferences)
M197400C	Test of quantification	Linearity, accuracy and precision verified over range from 50.0 to 25 000 ng/mL<br>230 nm kept as UV wavelength, 10 µL kept as injection volume
M197400D	Pre-qualification run	Results of linearity, accuracy and precision verified<br>Presence of an interference at the retention time of IS of about 8-10%, due to a possible contamination (not consistent with the previous results)<br>Method ready to be qualified, after changing all solvents (to avoid new contamination)

3. Final method

The developed method is presented in Table S2

Representative chromatograms of mitotane obtained from a blank sample, a calibration standard at the LLOQ (50.00 ng/mL) and a calibration standard at the ULOQ (10000 ng/mL) are presented in Table S4

The following tests were performed:

- Selectivity between mitotane and IS,
- Selectivity against endogenous interference and carry-over,
- Within-run accuracy and precision,
- Dilution integrity test.

4. Results

4.1. **Table S2 :** List of analytical runs

**Analytical run**	**Run description**	**Dataset retained**	**Run acceptance**	**Comments**
M197400A to M197400D	Development of the method	NA	Yes	/
M197400E	Selectivity against endogenous interferences and carry-over<br>Selectivity between Mitotane and IS<br>Within run accuracy and precision<br>Dilution integrity test	M197400E-03	No	Due to an interference at the retention time of the internal standard, the chromagraphic conditions were modified and the samples were re injected in the analytical run M197400E2
M197400E2	Selectivity against endogenous interferences and carry-over<br>Selectivity between Mitotane and IS<br>Within run accuracy and precision<br>Dilution integrity test	M197400E2-01	Yes	The volume in wells was too low for selectivity and accuracy tests, they had to be performed again.
M197400F	Selectivity against endogenous interferences and carry-over<br>Selectivity between Mitotane and IS<br>Within run accuracy and precision<br>Dilution integrity test	M197400F-01	No	2 Low QC samples and one High QC sample were out of specification.
M197400G	Assay of samples from rat 1,2,9,10,18 and 19 (all time points)	M197400G-04	Yes	/
M197400H	Assay of samples from rat 3 to 8, rat 11 to 16 (all time points) and rat 17 (predose and T30 min)	M197400H-02	Yes	/
M197400I	Assay of samples from rat 20 to 24 (all time points) and rat 17 T30 min 10-fold diluted<br>Selectivity against endogenous interferences and carry-over<br>Selectivity between Mitotane and IS	M197400I-03	Yes	/

Table S3: calibration curve

Analytical run	Slope	Intercept	r^2
M197400E2	0.000370798	-0.00159587	0.987478
M197400F	0.0000262039	0.00154145	0.996820
M197400G	0.000220936	-0.00113862	0.993109
M197400H	0.000241995	0.00191162	0.997995
M197400I	0.000251663	-0.00107532	0.996358

Representative calibration curve is presented in Figure S4 (File S1) (from analytical run M197400G).

Table S4: Concentrations (conc.) are expressed as ng/mL.

		Standard 1	Standard 2	Standard 3	Standard 4	Standard 5	Standard 6	Standard 7	Standard 8
**Analytical run**	**Nominal conc.**	**50.00**	**100.0**	**250.0**	**500.0**	**1000**	**2500**	**5000**	**10000**
M197400E2	conc.	45.73	116.2	NA[(1)]	516.0	1076	2309	4410	10090[(1)]
	% deviation	*-8.5*	*16.2*	*NA*	*3.2*	*7.6*	*-7.6*	*-11.8*	*0.9*
M197400F	conc.	48.12	-106.4	249.8	521.1	1056	2417	4901	9297
	% deviation	*-3.8*	*6.4*	*-0.1*	*4.2*	*5.6*	*-3.3*	*-2.0*	*-7.0*
M197400G	conc.	47.94	111.7	242.7	457.2	916.2	2574	5133	10660
	% deviation	*-4.1*	*11.7*	*-2.9*	*-8.6*	*-8.4*	*3.0*	*2.7*	*6.6*
M197400H	conc.	50.33	101.3	234.5	491.2	1023	2384	5169	10490
	% deviation	*0.7*	*1.3*	*-6.2*	*-1.8*	*2.3*	*-4.7*	*3.4*	*4.9*
M197400I	conc.	50.90	**52.33[(2)]**	236.8	452.0	1025	2554	5052	10740
	% deviation	*1.8*	***-47.7***	*-5.3*	*-9.6*	*2.5*	*2.1*	*1.0*	*7.4*

[(1)] Since volume remaining for Standards 3 and 8 was too low for the re-injection of analytical run M197400E2, there was no value for these two standards. The value from the SSTULOQ sample was used for calibration curve calculation.

[(2)] Out of acceptance criteria – not considered for calibration curve calculation.

4.2. **Table S5**: QC samples results

		Low QC	Mid QC	High QC
**Analytical run**	**Nominal concentration.**	**150**	**750**	**8000**
M197400E2	concentration.	127.2	731.0	7752
	% deviation	*-15.2*	*-2.5*	*-3.1*
	concentration.	150.9	585.1	7514
	% deviation	*0.6*	*-22.0*	*-6.1*
M197400F	concentration.	**78.66**(1)	793.7	**6017**(1)
	% deviation	***-47.6***	*5.8*	***-24.8***
	concentration.	**95.04**(1)	775.0	7456
	% deviation	***-36.6***	*3.3*	*-6.8*
M197400G	concentration.	142.1	695.9	8331
	% deviation	-5.3	-7.2	4.1
	concentration.	129.3	724.1	8205
	% deviation	*-13.8*	*-3.5*	*2.6*
M197400H	concentration.	129.0	739.4	7601
	% deviation	*-14.0*	*-1.4*	*-5.0*
	concentration.	147.7	729.9	7785
	% deviation	*-1.6*	*-2.7*	*-2.7*
	concentration.	141.0	774.6	8519
	% deviation	*-6.0*	*3.3*	*6.5*
	concentration.	147.8	829.8	**9744**(1)
	% deviation	*-1.5*	*10.6*	***21.8***
M197400I	concentration.	137.8	664.2	7493
	% deviation	*-8.1*	*-11.4*	*-6.3*
	concentration.	136.6	690.7	7330
	% deviation	*-8.9*	*-7.9*	*-8.4*

(1) Out of acceptance criteria

4.3. Selectivity between mitotane and IS

4.3.1. Procedure and acceptance criteria

The selectivity of the method for the assay of mitotane was verified against the internal standard by analyzing matrix samples spiked with individual solution of mitotane or IS.

The analytical run included:

- The system suitability test (SST),
- 1 matrix sample spiked with mitotane at the ULOQ (1 injection),
- 1 matrix sample spiked with IS at the working concentration (1 injection),
- 1 matrix sample spiked with mitotane at the LLOQ and IS at the working concentration (3 injections).

The selectivity of the method between mitotane and IS was demonstrated when the injection of matrix samples spiked with mitotane at the ULOQ or the injection of a matrix samples spiked with IS at the working concentration did not induce any significant signal at the retention time of mitotane or IS.

If present, the interference between mitotane and IS was calculated as follows:

$$\text{Interference}_{\text{Analyte}}\,(\%) = \frac{\text{Interference peak area of the analyte}}{\text{Mean peak area of the analyte at the LLOQ (n = 3)}} \times 100$$

If present, the interference between the IS and mitotane was calculated as follows:

$$\text{Interference}_{\text{IS}}\,(\%) = \frac{\text{Interference peak area of IS}}{\text{Mean Peak Area of IS at the working concentration (n = 3)}} \times 100$$

The selectivity between mitotane and IS was demonstrated if the interference was < 20% for mitotane and < 5% for the IS.

4.3.2. **Table S6:** Results of selectivity between Mitotane and IS

ULOQ in the sample	10000 ng/mL	IS conc. in the sample		5000 ng/mL	
**Compound in the sample**	**Sample ID**	**Mitotane**		**Mitotane-d5 (IS)**	
		**Peak area**	**% Interf**	**Peak area**	**% Interf**
Mitotane	I_ULOQselectivity_32	/	/	1.59	0.2
IS	I_Zero_11	1.28	14.9	/	/
**Acceptance criteria**		**< 20.0 % of the mean peak area of Mitotane at the LLOQ**		**< 5.0 % of the mean peak area of IS at 5000 ng/mL**	

LLOQ in the sample	50.00 ng/mL	
**Sample ID**	**Peak area**	
	**Mitotane**	**IS**
I_LLOQselectivity_33	8.65	827.18
I_LLOQselectivity_34	8.76	827.58
I_LLOQselectivity_35	8.39	828.65
**Mean area**	**8.60**	**827.80**

4.4. Selectivity against endogenous interference and carry-over

4.4.1. Procedure and acceptance criteria

The selectivity of the method against endogenous interference was verified by analysis of 3 blank samples prepared from 3 different sources of control matrix (3 different animals).

Three matrix samples spiked with mitotane at the LLOQ and IS at the working concentration were extracted in order to determine the reference peak areas of mitotane at the LLOQ and of IS.

The carry-over was checked by re-injecting the blank samples after a sample at the ULOQ.

The analytical run included:

- The system suitability test,
- 1 blank sample, 1 zero sample,
- 1 calibration curve,
- 3 LLOQ Val samples,
- 6 blank samples (first injection for selectivity) prepared with control matrices from 6 different sources,
- 1 ULOQ sample inserted before each of the 6 blank samples (second injection for carry-over),
- 2 QCs per concentration level (Low, Mid and High).

Acceptance criteria for the LLOQ Val samples: The percentage error between the mean measured concentration (n = 3) and the theoretical concentration had to be within ± 20 %.

Acceptance criteria for each ULOQ sample: The percentage error between the individual measured concentration and the theoretical concentration had to be upper than -20 %. All the ULOQ samples had to meet this criterion.

No significant interference had to be present at the retention time window of mitotane and IS for the 6 blank samples for the selectivity test and for the carry-over test.

If present, the interference was calculated as follows:

$$\text{Interference}_{\text{Analyte}}\ (\%) = \frac{\text{Interference peak area}}{\text{Mean peak area of analyte at the LLOQ (n = 3)}} \times 100$$

$$\text{Interference}_{\text{IS}}\ (\%) = \frac{\text{Interference peak area}}{\text{Mean peak area of IS at the working concentration (n = 3)}} \times 100$$

The selectivity (1st injection of the 6 blank samples) and the absence of carry-over (2nd injection of the 6 blank samples) was demonstrated when the interference was < 20% for mitotane and < 5% for the IS.

4.4.2. Results

4.4.2.1. Table S7: Selectivity against endogenous interferences

Blank matrix batch	Sample ID	Mitotane		4,4'-DDT (IS)	
		Peak area	% Interf	Peak area	% Interf
T1719	I_Blank T1719_5	1.68	19.5	2.43	0.3
T1722	I_Blank T1722_6	0.45	5.2	5.12	0.6
T19295	I_Blank T19295_7	1.45	16.9	5.43	0.7
T19296	I_Blank T19296_8	ND	NA	2.29	0.3
T19297	I_Blank T19297_9	ND	NA	2.91	0.4
T19298	I_Blank T19298_10	1.55	18.0	36.06	4.4
**Acceptance criteria**		**< 20.0 % of the mean peak area of Mitotane at the LLOQ**		**< 5.0 % of the mean peak area of IS at 5000 ng/mL**	

LLOQ

Theoretical concentration (ng/mL)	Sample ID	Peak area		Measured concentration (ng/mL)
		Mitotane	IS	
**50.00**	I_LLOQselectivity_33	8.65	827.18	45.83
	I_LLOQselectivity_34	8.76	827.58	46.34
	I_LLOQselectivity_35	8.39	828.65	44.51
	**Mean**	**8.60**	**827.80**	**45.56**
			**% Diff.**	**-8.9**

	**Acceptance criteria: Diff. within ± 20.0 %**

ND: not detected
NA: Not applicable

4.4.2.2. **Table S8:** Carry-over

**Blank matrix batch**	**Sample ID**	**Mitotane**		**4,4'-DDT (IS)**	
		**Peak area**	**% Interf**	**Peak area**	**% Interf**
T1719	I_Blank T1719_21	1.27	14.8	3.38	0.4
T1722	I_Blank T1722_23	ND	NA	3.65	0.4
T19295	I_Blank T19295_25	1.49	17.3	3.89	0.5
T19296	I_Blank T19296_27	0.82	9.5	1.3	0.2
T19297	I_Blank T19297_29	ND	NA	2.55	0.3
T19298	I_Blank T19298_31	1.4	16.3	39.27	4.7
**Acceptance criteria**		**< 20.0 % of the mean peak area of Mitotane at the LLOQ**		**< 5.0 % of the mean peak area of IS at 5000 ng/mL**	

LLOQ

**Theoretical concentration (ng/mL)**	**Sample ID**	**Peak area**		**Measured concentration (ng/mL)**
		**Mitotane**	**IS**	
**50.00**	I_LLOQselectivity_33	8.65	827.18	45.83
	I_LLOQselectivity_34	8.76	827.58	46.34
	I_LLOQselectivity_35	8.39	828.65	44.51
	**Mean**	**8.60**	**827.80**	45.56
			**% Diff.**	-8.9
			**Acceptance criteria: Diff. within ± 20.0 %**	

ULOQ

**Theoretical concentration (ng/mL)**	**Sample ID**	**Measured concentration (ng/mL)**	**% Diff.**
**10000**	I_ULOQVal1_20	8639	-13.6
	I_ULOQVal1_22	8643	-13.6
	I_ULOQVal1_24	8610	-13.9
	I_ULOQVal2_26	9066	-9.3
	I_ULOQVal2_28	9029	-9.7
	I_ULOQVal2_30	9052	-9.5

Acceptance criteria: Diff. within ± 20.0 %

ND: not detected
NA: Not applicable

4.5. Precision and accuracy

4.5.1. Procedure and acceptance criteria

The within-run accuracy and precision were evaluated at 4 concentration levels (LLOQ, Low, Mid and High) after replicate analysis of Val samples, n = 5 per concentration level.

The analytical runs included:

- The system suitability test,
- 1 blank sample,
- 1 zero sample,
- 1 calibration curve,
- 5 Val samples per concentration level (LLOQ, Low, Mid and High).

The within-run accuracy was assessed as the percentage error (bias) between the mean measured concentration and the corresponding theoretical concentration, as follows:

$$\%\ \text{Error}_{\text{Within-run}} = \frac{\text{Mean measured conc. at X level (n = 5)} - \text{Theoretical conc.}}{\text{Theoretical conc.}} \times 100$$

The acceptable within-run percentage error had to be within ±20%.

The within-run precision was assessed as the coefficient of variation taking into account the 5 replicates, as follows:

$$\text{CV}_{\text{Within-run}}\ (\%) = \frac{\text{Standard Deviation}}{\text{Mean measured conc. at X level (n = 5)}} \times 100$$

The highest acceptable coefficient of variation for the within-run precision was 20%.

4.5.2. **Table S9 :** Results of precision and accuracy

	LLOQ Val (50.00 ng/mL)		Low Val (150.0 ng/mL)		Mid Val (750.0 ng/mL)		High Val (8000 ng/mL)	
**Analytical run ID**	**Sample ID**	**Measured concentration (ng/mL)**	**Sample ID**	**Measured concentration (ng/mL)**	**Sample ID**	**Measured concentration (ng/mL)**	**Sample ID**	**Measured concentration (ng/mL)**
M197400E2	E2_LLOQVal1_32	49.69	E2_LowVal1_37	127.2	E2_MidVal1_42	731.0	E2_HighVal1_52	7752
	E2_LLOQVal2_33	44.55	E2_LowVal2_38	153.7	E2_MidVal2_43	500.2	E2_HighVal2_53	8537
	E2_LLOQVal3_34	52.84	E2_LowVal3_39	135.0	E2_MidVal3_44	781.5	E2_HighVal3_54	7885
	E2_LLOQVal4_35	49.21	E2_LowVal4_40	152.2	E2_MidVal4_45	732.5	E2_HighVal4_55	7612
	E2_LLOQVal5_36	53.39	E2_LowVal5_41	150.9	E2_MidVal5_46	585.1	E2_HighVal5_56	7514
**Within-run mean**		**49.94**		**143.8**		**666.1**		**7860**
**Within-run %Error**		**-0.1**		**-4.1**		**-11.2**		**-1.8**
**Within-run CV (%)**		**7.1**		**8.3**		**17.8**		**5.1**
**Acceptance criteria**	**Within-run %Error: within ± 20.0%<br>Within-run CV : ≤ 20.0%**							

4.6. dilution integrity

4.6.1. Procedure and acceptance criteria

The dilution test was conducted on a high level of mitotane at 5 times the ULOQ (Dil Val sample.

The Dil Val sample was 10-fold diluted with the control matrix (5 replicates), then extracted using the current method and assayed by LC-MS/MS.

The analytical run included:

- The system suitability test,
- 1 blank sample, 1 zero sample,
- 1 calibration curve,
- 5 Dil Val samples,
- 2 QC samples per concentration level (Low, Mid and High).

The dilution accuracy was expressed as the following percentage error:

$$\% \text{ Error} = \frac{\text{Mean measured conc. of Dil Val samples (n = 5)} - \text{Theoretical conc.}}{\text{Theoretical conc.}} \times 100$$

The acceptable percentage error had to be be within ±20 %.

The precision was expressed as the coefficient of variation (CV) taking into account the variability of the 5 Dil Val samples as follows:

$$\text{CV (\%)} = \frac{\text{Standard Deviation}}{\text{Mean measured conc. of Dil Val samples (n = 5)}} \times 100$$

The highest acceptable coefficient of variation was 20 %.

4.6.2. **Table S10** : Results

	Dilution factor:		10			
**Sample ID**	**Theoretical concentration (pg/mL)**	**Measured concentration (pg/mL)**	**n**	**Mean (pg/mL)**	**Error (%)**	**CV (%)**
E2_DilVal1_47		45110				
E2_DilVal2_48		41080				
E2_DilVal3_49	50000	43880	5	44620	-10.8	7.7
E2_DilVal4_50		42860				
E2_DilVal5_51		50150				
	**Acceptance criteria:**		**Error within ± 20.0 % and CV ≤ 20.0 %**			

4.7. Mitotane concentrations

Specimens were analyzed in singlicate.

Concentrations in plasma was expressed in nanograms per milliliter (ng/mL) with 4 significant figures.

Data below the LLOQ were reported as ‘BLQ’.

Measured concentrations of mitotane are presented in Table S11

Appendix 1: Representative chromatograms of mitotane in rat plasma

Figure S1 : Blank sample

Name: I_Blank T19297_9, Date: 19-Dec-2019, Time: 02:33:11, ID: , Description: Blank T19297

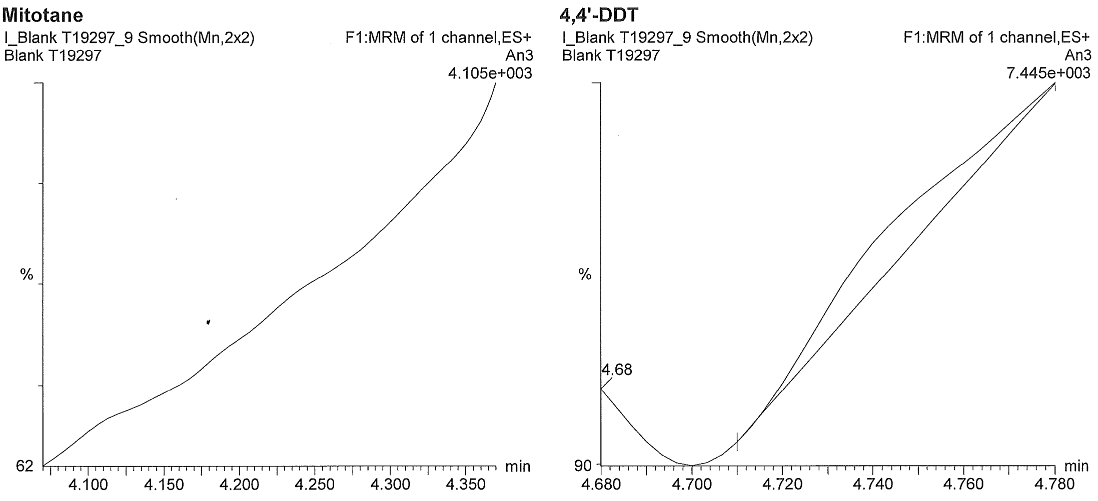

# Name	RT	Area	Flags
1 Mitotane			
2 4,4'-DDT	4.78	2.91	MM

Figure S2 : Control matrix spiked with 50.00 ng/mL of mitotane (LLOQ)

Name: G_Standard1_7, Date: 16-Dec-2019, Time: 19:47:34, ID: , Description: Standard1

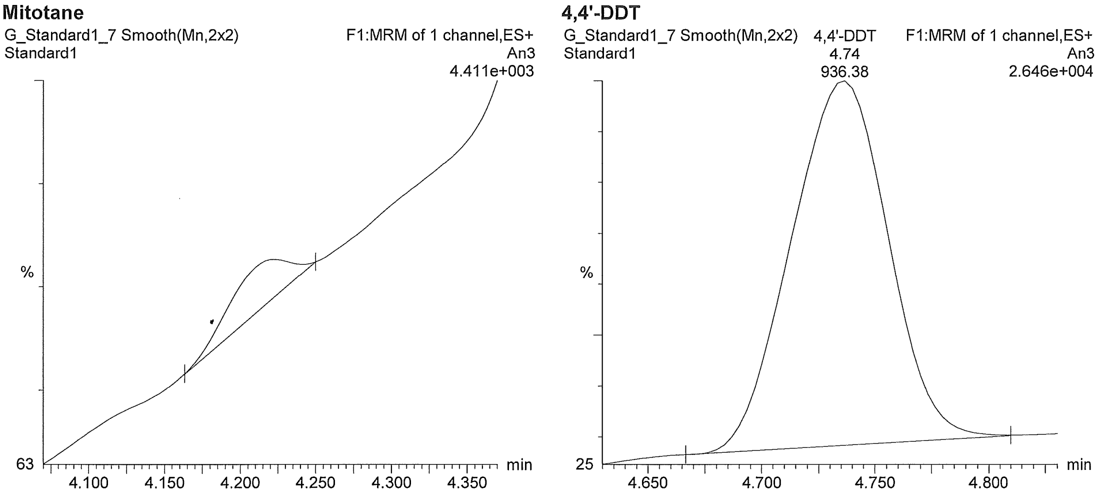

# Name	RT	Area	Flags
1 Mitotane	4.22	8.85	MM
2 4,4'-DDT	4.74	936.38	MM

Figure S3: Control matrix spiked with 10000 ng/mL of mitotane (ULOQ)

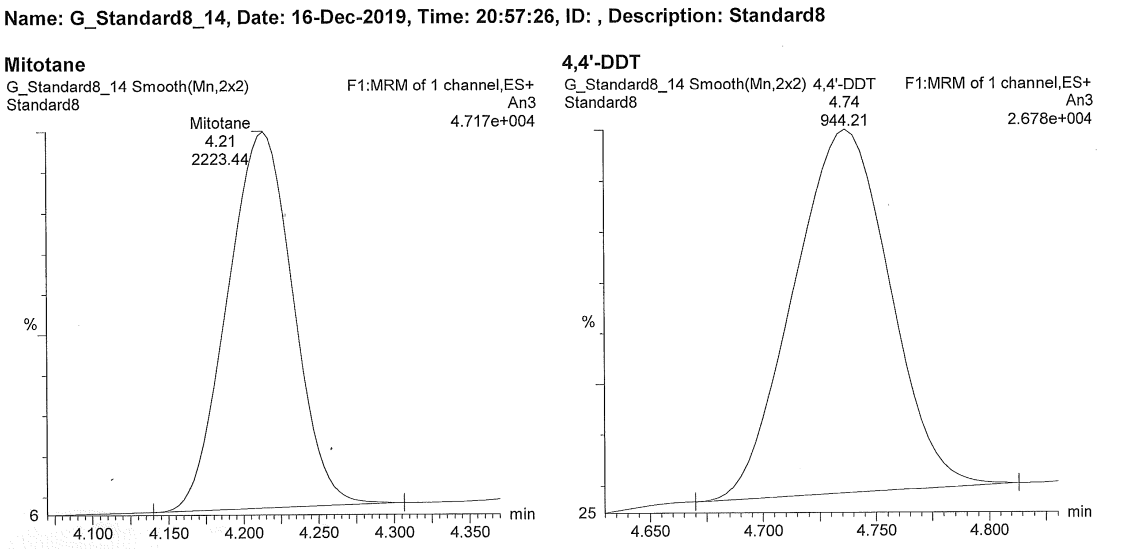

# Name	RT	Area	Flags
1 Mitotane	4.21	2223.44	bb
2 4,4'-DDT	4.74	944.21	MM

Appendix 2:

Figure S4: Calibration curve for mitotane for the analytical run M197400G

Quantify Calibration Report MassLynx 4.2 SCN986 Page 1 of 1
HPLC54 - MassLynx 4.2 - TargetLynx XS 4.2

Dataset: D:\A197400.PRO\CurveDB\M197400G_04.qld
Last Altered: Tuesday, January 07, 2020 14:20:01 Romance Standard Time
Printed: At Tuesday, January 07, 2020 14:20:36 Romance Standard Time
By FTN-DES-748\desmartin_e (Desmartin Emmanuel)

Method: D:\A197400.PRO\MethDB\M197400_03.mdb 17 Dec 2019 08:35:36
Calibration: 07 Jan 2020 14:16:38

Compound name: Mitotane
Correlation coefficient: r = 0.996549, r^2 = 0.993109
Calibration curve: 0.000220936 * x + -0.00113862
Response type: Internal Std (Ref 2), Area * (IS Conc. / IS Area)
Curve type: Linear, Origin: Exclude, Weighting: 1/x^2, Axis trans: None

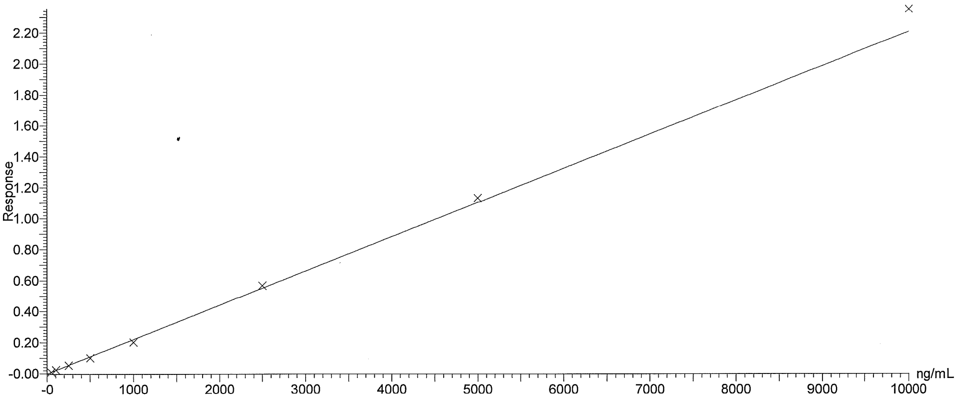

Compound name: 4,4'-DDT
Response Factor: 944.762
RRF SD: 45.0553, % Relative SD: 4.76896
Response type: External Std, Area
Curve type: RF

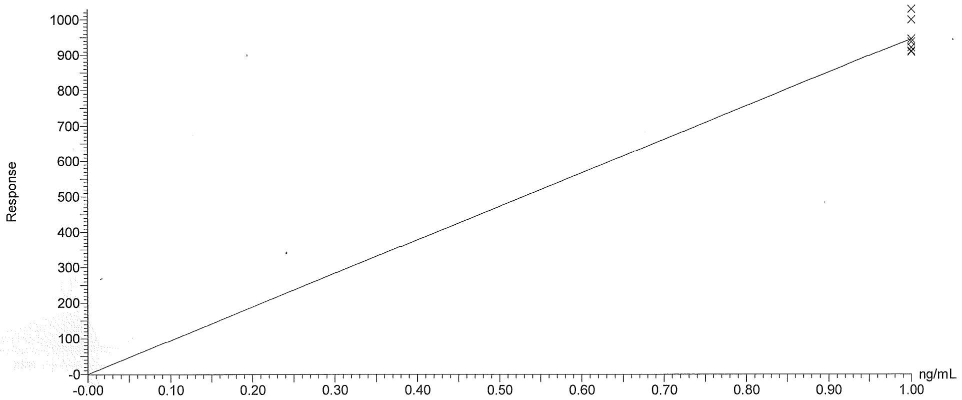

File S2 : Individual mitotane plasma concentrations in rats

Table S11: Individual mitotane plasma concentrations in rats

Animal ID	Group	Administered formulation	Target dose administered (mg/kg)	Actual dose administered (mg/kg)	Theoretical Sampling Time (h)	Sampling Time (h)	Plasma conc, ng/mL
1	1	Lysodren	100	99.326	0	0	BLQ
					0.5	0.5	BLQ
					1	1	280.6
					2	2.017	396.5
					3	3	379.6
					4	4	444.6
					5	5	497.5
					6	6	311.2
					9	9.017	98.96
					12	12.033	BLQ
					24	24	BLQ
					48	48.017	BLQ
2	1	Lysodren	100	95.834	0	0	BLQ
					0.5	0.5	64.82
					1	1	202.0
					2	2	514.1
					3	3	547.7
					4	4	733.0
					5	5	669.3
					6	6	443.5
					9	9	96.45
					12	12.017	52.00
					24	24	BLQ
					48	48	BLQ
3	1	Lysodren	100	100.438	0	0	BLQ
					0.5	0.5	57.81
					1	1	474.4
					2	2	1198
					3	3	1399
					4	4	1195
					5	5	891.0
					6	6	657.0
					9	8.983	152.3
					12	12	96.33
					24	24	BLQ
					48	48	BLQ

Table S11: Individual mitotane plasma concentrations in rats (continued)

Animal ID	Group	Administered formulation	Target dose administered (mg/kg)	Actual dose administered (mg/kg)	Theoretical Sampling Time (h)	Sampling Time (h)	Plasma conc, ng/mL
4	1	Lysodren	100	97.828	0	0	BLQ
					0.5	0.55	75.52
					1	1	101.9
					2	2.017	981.9
					3	3	1974
					4	4	2467
					5	5	2551
					6	6	2183
					9	8.983	928.4
					12	12	645.7
					24	24	108.1
					48	48	52.53
5	1	Lysodren	100	98.483	0	0	BLQ
					0.5	0.5	173.6
					1	0.983	755.9
					2	1.983	1640
					3	2.983	2321
					4	3.983	3684
					5	4.983	2068
					6	5.983	1872
					9	8.983	535.0
					12	11.983	278.4
					24	23.983	61.24
					48	47.983	BLQ
6	1	Lysodren	100	99.407	0	0	BLQ
					0.5	0.5	57.93
					1	1	208.6
					2	2	817.2
					3	3	1302
					4	4	1058
					5	5	1301
					6	6	895.2
					9	9	223.2
					12	12	152.6
					24	24	BLQ
					48	47.983	BLQ

Table S11: Individual mitotane plasma concentrations in rats (continued)

Animal ID	Group	Administered formulation	Target dose administered (mg/kg)	Actual dose administered (mg/kg)	Theoretical Sampling Time (h)	Sampling Time (h)	Plasma conc, ng/mL
7	1	Lysodren	100	99.167	0	0	BLQ
					0.5	0.5	BLQ
					1	1	211.5
					2	2	813.5
					3	3	1212
					4	4	1175
					5	5	755.2
					6	6	647.6
					9	9	343.4
					12	12	161.7
					24	24	BLQ
					48	48	BLQ
8	1	Lysodren	100	95.985	0	0	BLQ
					0.5	0.5	135.4
					1	1	406.7
					2	2	1059
					3	3	1552
					4	4	1443
					5	5	1186
					6	6	1212
					9	9	417.3
					12	12	328.9
					24	24	57.33
					48	48.017	BLQ
9	2	Mito F1	100	94.871	0	0	BLQ
					0.5	0.5	750.9
					1	1	1184
					2	2.017	1725
					3	3	3641
					4	4	3871
					5	5	3567
					6	6	3318
					9	9	1427
					12	12	761.3
					24	24	103.9
					48	48	54.92

Table S11: Individual mitotane plasma concentrations in rats (continued)

Animal ID	Group	Administered formulation	Target dose administered (mg/kg)	Actual dose administered (mg/kg)	Theoretical Sampling Time (h)	Sampling Time (h)	Plasma conc, ng/mL
10	2	Mito F1	100	92.998	0	0	BLQ
					0.5	0.5	283.0
					1	1.017	451.1
					2	2	687.1
					3	3	1731
					4	4	2708
					5	5	3513
					6	6	4350
					9	9	2087
					12	12	1122
					24	24.017	116.1
					48	48	69.74
11	2	Mito F1	100	98.196	0	0	BLQ
					0.5	0.517	257.1
					1	1.017	456.5
					2	2	1433
					3	3	2871
					4	4	4243
					5	5	3543
					6	6	3958
					9	9	2331
					12	12	1423
					24	24	216.6
					48	48	89.89
12	2	Mito F1	100	96.259	0	0	BLQ
					0.5	0.5	1099
					1	1.017	2175
					2	2	3595
					3	3	4533
					4	4	6991
					5	5	3815
					6	6	4066
					9	9	1264
					12	12	377.8
					24	24	125.5
					48	48.017	56.68

Table S11: Individual mitotane plasma concentrations in rats (continued)

Animal ID	Group	Administered formulation	Target dose administered (mg/kg)	Actual dose administered (mg/kg)	Theoretical Sampling Time (h)	Sampling Time (h)	Plasma conc, ng/mL
13	2	Mito F1	100	96.961	0	0	BLQ
					0.5	0.5	624.7
					1	1.017	857.5
					2	2	1008
					3	3	3012
					4	4	7815
					5	5	4417
					6	6	4276
					9	9	1388
					12	12	793.4
					24	24.017	182.9
					48	48	80.30
14	2	Mito F1	100	97.33	0	0	BLQ
					0.5	0.5	614.6
					1	1	1451
					2	2	2866
					3	3	4439
					4	4	4596
					5	5	5267
					6	6	4199
					9	9	2083
					12	12	974.2
					24	24	168.8
					48	48	65.30
15	2	Mito F1	100	97.223	0	0	BLQ
					0.5	0.5	701.7
					1	1	1934
					2	2	3418
					3	3	5895
					4	4	5715
					5	5	4562
					6	6	3487
					9	9	1554
					12	12	533.4
					24	24	126.3
					48	48.017	82.80

Table S11: Individual mitotane plasma concentrations in rats (continued)

Animal ID	Group	Administered formulation	Target dose administered (mg/kg)	Actual dose administered (mg/kg)	Theoretical Sampling Time (h)	Sampling Time (h)	Plasma conc, ng/mL
16	2	Mito F1	100	94.193	0	0	BLQ
					0.5	0.5	1127
					1	1	3018
					2	2	5591
					3	3	7254
					4	4	6319
					5	5	6959
					6	6	4967
					9	9	2256
					12	12	1422
					24	24	237.5
					48	47.983	136.6
17	3	Mito F2	100	97.14	0	0	BLQ
					0.5	0.5	14300
					1	0	NA
					2	0	NA
					3	0	NA
					4	0	NA
					5	0	NA
					6	0	NA
					9	0	NA
					12	0	NA
					24	0	NA
					48	0	NA
18	3	Mito F2	100	96.274	0	0	BLQ
					0.5	0.517	1326
					1	1	2358
					2	2	4534
					3	3	5451
					4	4	5590
					5	5	4336
					6	6	4092
					9	9	945.3
					12	12	421.8
					24	24	129.5
					48	47.967	BLQ

Table S11: Individual mitotane plasma concentrations in rats (continued)

Animal ID	Group	Administered formulation	Target dose administered (mg/kg)	Actual dose administered (mg/kg)	Theoretical Sampling Time (h)	Sampling Time (h)	Plasma conc, ng/mL
19	3	Mito F2	100	92.784	0	0	BLQ
					0.5	0.5	2184
					1	1	3961
					2	2	4643
					3	3	5048
					4	4	5235
					5	5	3151
					6	6	2567
					9	9	1227
					12	12	590.0
					24	24	134.7
					48	47.933	BLQ
20	3	Mito F2	100	97.104	0	0	BLQ
					0.5	0.5	1406
					1	1.017	2567
					2	2	4241
					3	3	7297
					4	4	7110
					5	5	5777
					6	6	4915
					9	9	1043
					12	12	563.0
					24	24	142.4
					48	47.9	62.35
21	3	Mito F2	100	95.295	0	0	BLQ
					0.5	0.5	677.7
					1	1	1969
					2	2	3402
					3	3	5325
					4	4	4555
					5	5	2675
					6	6	2068
					9	9	561.1
					12	12	392.2
					24	24	103.7
					48	47.883	59.42

Table S11: Individual mitotane plasma concentrations in rats (end)

Animal ID	Group	Administered formulation	Target dose administered (mg/kg)	Actual dose administered (mg/kg)	Theoretical Sampling Time (h)	Sampling Time (h)	Plasma conc, ng/mL
22	3	Mito F2	100	96.967	0	0	BLQ
					0.5	0.5	970.2
					1	1	2063
					2	2	3074
					3	3	3890
					4	4	3709
					5	5	2997
					6	6	3819
					9	9	1220
					12	12	829.2
					24	24	149.9
					48	47.867	91.89
23	3	Mito F2	100	94.754	0	0	BLQ
					0.5	0.5	BLQ
					1	1.017	BLQ
					2	2	58.84
					3	3	122.9
					4	4	433.3
					5	5	1270
					6	6	1300
					9	9	1059
					12	12	1361
					24	24	167.5
					48	47.867	70.05
24	3	Mito F2	100	93.941	0	0	BLQ
					0.5	0.517	143.8
					1	1	406.1
					2	1.983	1375
					3	3	2655
					4	4	2225
					5	5	4762
					6	6	5678
					9	9	1493
					12	12	795.0
					24	24	148.7
					48	47.817	99.78
